# Development and validation of an abnormality-derived deep-learning diagnostic system for major respiratory diseases

**DOI:** 10.1038/s41746-022-00648-z

**Published:** 2022-08-23

**Authors:** Chengdi Wang, Jiechao Ma, Shu Zhang, Jun Shao, Yanyan Wang, Hong-Yu Zhou, Lujia Song, Jie Zheng, Yizhou Yu, Weimin Li

**Affiliations:** 1 Department of Respiratory and Critical Care Medicine, Med-X Center for Manufacturing, Frontiers Science Center for Disease-related Molecular Network, West China Hospital, West China School of Medicine, Sichuan University, Chengdu, China; 2 AI Lab, Deepwise Healthcare, Beijing, China; 3 Nursing Key Laboratory of Sichuan Province, National Clinical Research Center for Geriatrics, and Science and Technology Department, West China Hospital, Sichuan University, Chengdu, China; 4 Department of Computer Science, The University of Hong Kong, Pokfulam, Hong Kong

**Keywords:** Classification and taxonomy, Respiratory tract diseases

## Abstract

Respiratory diseases impose a tremendous global health burden on large patient populations. In this study, we aimed to develop DeepMRD^TR^, a deep learning-based medical image interpretation system for the diagnosis of major respiratory diseases based on the automated identification of a wide range of radiological abnormalities through computed tomography (CT) and chest X-ray (CXR) from real-world, large-scale datasets. DeepMRD^TR^ comprises four networks (two CT-Nets and two CXR-Nets) that exploit contrastive learning to generate pre-training parameters that are fine-tuned on the retrospective dataset collected from a single institution. The performance of DeepMRD^TR^ was evaluated for abnormality identification and disease diagnosis on data from two different institutions: one was an internal testing dataset from the same institution as the training data and the second was collected from an external institution to evaluate the model generalizability and robustness to an unrelated population dataset. In such a difficult multi-class diagnosis task, our system achieved the average area under the receiver operating characteristic curve (AUC) of 0.856 (95% confidence interval (CI):0.843–0.868) and 0.841 (95%CI:0.832–0.887) for abnormality identification, and 0.900 (95%CI:0.872–0.958) and 0.866 (95%CI:0.832–0.887) for major respiratory diseases’ diagnosis on CT and CXR datasets, respectively. Furthermore, to achieve a clinically actionable diagnosis, we deployed a preliminary version of DeepMRD^TR^ into the clinical workflow, which was performed on par with senior experts in disease diagnosis, with an AUC of 0.890 and a Cohen’s *k* of 0.746–0.877 at a reasonable timescale; these findings demonstrate the potential to accelerate the medical workflow to facilitate early diagnosis as a triage tool for respiratory diseases which supports improved clinical diagnoses and decision-making.

## Introduction

Respiratory diseases are among the leading causes of morbidity and mortality, posing a significant burden worldwide^1^. Globally, chronic respiratory diseases impacted a large patient group accounting for 7.4% of the world’s population and led to 7.0% of total all-cause deaths^2^. Lower respiratory infections kill millions of people annually^3^, and for example, the COVID-19 pneumonia pandemic alone caused more than two million deaths during the 1st year since the outbreak^4^. Lung cancer is the leading cause of cancer-related mortality, with the 5- year survival rate of 10-20% in most countries^5^. Tuberculosis (TB) is the most common lethal infectious disease, ranking above the human immunodeficiency virus/acquired immunodeficiency syndrome since 2007^6^. These mentioned above are considered the most important lung diseases worldwide from a prevalence standpoint, according to the Forum of International Respiratory Societies^1^. Respiratory diseases, which impose an immense and persistent burden on the health care system worldwide, are intrinsically difficult to diagnose, mainly due to the unavailability of necessary and important diagnostic equipment in remote areas or resource-constrained settings. It is an urgent need to develop a new tool to accelerate homogenization of the diagnosis of respiratory diseases, particularly in areas where medical resources are unevenly distributed or scarce in China.

Radiology plays an indispensable role in the screening, triaging, and diagnosis of various respiratory diseases. Chest radiography, often known as chest X-ray (CXR), is the most commonly used first-line investigative technique for disease evaluation^7^. Computed tomography (CT), which can generate three-dimensional (3D) volumes and offer more precise information on pathologies than CXR images, is also a mainstay of medical imaging strategies for thoracic disease diagnosis^8^. Although these techniques can capture digital texture invisible to human eyes, the accurate diagnosis is still challenging owing to the lack of interobserver agreement in radiological evaluation^9^. There are many chest abnormalities; the co-occurrence of multiple abnormalities is frequently observed in the same imaging modality, and the same pathology may disperse in various sites in one scan^8^. A wide variety of chest abnormalities pose a huge challenge to the accurate diagnosis and treatment of respiratory diseases. Therefore, improving the use of bulk radiological images has been of paramount importance and enormous value.

Recent exciting developments in artificial intelligence (AI) have opened up a new chapter in medical image analysis^10–15^. Prior studies have demonstrated the general applicability of deep learning methods in classifying age-related macular degeneration and diabetic macular edema, grading diabetic retinopathy, identifying skin cancer subtypes, detecting breast cancer metastasis, and triaging critical findings in head CT abnormalities^16–20^. Deep learning algorithms have also been trained and developed to identify thoracic abnormalities or diseases based on either CXR or CT images^8,21^. Previously, we developed deep learning-based medical image interpretation systems for the early diagnosis of COVID-19 pneumonia and the identification of malignant lung nodules, which demonstrated the promising applicability in both acute and non-acute respiratory disease care settings^22–24^. However, such established AI systems simply focus on one disease differential diagnosis or single disease binary diagnosis, limiting their clinical applicability and generalizability in the real-world routine practice with a variety of respiratory conditions.

There have been several prior studies on thoracic disease diagnosis and abnormality detection systems^8,21–25^. Hwang et al.^21^ developed a deep learning-based algorithm that could classify four major thoracic diseases, including pulmonary malignant tumors, active TB, pneumonia, and pneumothorax, based on chest radiographs. In that system, the algorithm covered only four categories of thoracic diseases, which in fact only took a small proportion of clinically relevant diseases. Another drawback was that the datasets performed for validation were experimentally designed, and only represented one single target disease, hence were distinct from real-world conditions. Our team previously developed an AI system for the diagnosis of common lung diseases using CXR images^23^. However, the approach did not distinguish between thoracic diseases and chest abnormalities. Furthermore, CXR is limited in distinguishing multiple target diseases in real-world situations because of the inferior presentation of less-well-defined tissue structures and lack of three-dimensional information; thus, the adoption of CT scans is necessary for the diagnosis of several specific diseases. Recent CT-based approaches for detecting abnormalities could extract features on slices and then fuse them into volume levels, which raised the demand for more contextual information on 3D extraction^8^. Although different models have been proposed for the detection of lung disorders, a fully automatic analysis pipeline that is robust in diverse CT/CXR imaging conditions and satisfies the requirements needed for real-world clinical deployment is still lacking. There are three main challenges of the large-scale multi-label classification of two- and three-dimensional images: difficulties to obtain substantial high-quality labels, obstacles to accurately identify multiple abnormalities, and challenges of developing large-scale multi-label multi-task diagnostic models.

In the context of precision medicine, we aimed at generating an AI-based automatic analysis pipeline to empower precise abnormality identification and accurate disease diagnosis in the respiratory field. Here, we developed the DeepMRD^TR^ model based on the deep-learning algorithms to address the aforementioned realistic clinical application and technological issues through real-world large-scale CT scans and CXR images. To verify the generalizability and robustness of the DeepMRD^TR^ system, we validated the system in an external dataset collected from another institution. We compared the performance of AI system on CT and CXR images to enable actual deployment in the scenario where CT devices are less available. Further we deployed a preliminary version of the AI system into the clinical workflow to demonstrate the feasibility of incorporating our AI system into real-life clinical workflows in a human + AI fashion with advantages on time consumption and prediction accuracy. Therefore, our model will hopefully aid junior physicians in developing their competence, and senior physicians in improving their efficiency.

## Results

### Data sources and patient characteristics

We constructed a large chest scan dataset from two primary subsets: (1) one from West China Hospital (WCH) for training and internal testing and (2) the other from Chengdu ShangJin Nanfu Hospital (CSJH) for external validation to evaluate the model’s generalizability and robustness to an unrelated population. We hypothesized that training the system with image input might only be associated with disease textures that manifested at different time points after hospital admission. We used the initial examination from each hospital admission that had not received treatment for this condition. The CT dataset from the two hospitals comprised 228,563 CT volumes (*n* = 52,200), including 191,333 (*n* = 43,966) CT volumes chosen at random for developing and internal testing (WCH) the AI system, and the other 37,230 (*n* = 8234) for external validation (CSJH). The CXR dataset contained 129,319 images (*n* = 67,611) for the same tasks, among which 125,599 CXR images (*n* = 64,451) were used for training and internal testing and an additional 3720 images (*n* = 3160) were used for external validation. Patient demographics and characteristics of each critical finding from scans of the training, testing and validation datasets are summarized in Table 1, and the flow of study design is shown in Supplementary Fig. 1.

**Table 1:** Summary of training, internal testing and external validation datasets.

Demographics	CT dataset (*n* = 52,200)			CXR dataset (*n* = 67,611)		
	Training cohort (*n* = 34,533)	Internal testing cohort (*n* = 9433)	External validation cohort (*n* = 8234)	Training cohort (*n* = 45,466)	Internal testing cohort (*n* = 18,985)	External validation cohort (*n* = 3160)
Age (years)	56.702 ± 15.946	54.465 ± 15.833	49.653 ± 14.484	51.270 ± 18.613	50.465 ± 19.863	47.965 ± 21.461
Sex (male)	19,338 (56.0%)	5207 (55.2%)	4399 (53.4%)	26,675 (58.7%)	10,783 (56.8%)	1709 (54.1%)
In-hospital	147,754	43,579	37,230	86,647	38,952	3720
Diseases						
Bronchiectasis	10,319 (7.0%)	2820 (6.5%)	1216 (3.3%)	1414 (1.6%)	769 (2.0%)	26 (0.7%)
COPD	24,918 (16.9%)	6591 (15.1%)	5105 (13.7%)	8724 (10.1%)	2903 (7.5%)	82 (2.2%)
ILD	8829 (6.0%)	3068 (7.0%)	325 (0.9%)	670 (0.8%)	740 (1.9%)	8 (0.2%)
Lung cancer	16,184 (11.0%)	4860 (11.2%)	3130 (8.4%)	17,419 (20.1%)	9001 (23.1%)	887 (23.8%)
Pleural effusion	41,600 (28.2%)	10,686 (24.5%)	5846 (15.7%)	27,107 (31.3%)	12,910 (33.1%)	790 (21.2%)
Pneumonia	92,004 (62.3%)	27,334 (62.7%)	8756 (23.5%)	35,769 (41.3%)	20,476 (52.6%)	373 (10.0%)
Pneumothorax	4795 (3.2%)	1513 (3.5%)	853 (2.3%)	10,135 (11.7%)	4021 (10.3%)	736 (19.8%)
TB	17,051 (11.5%)	3988 (9.2%)	592 (1.6%)	1698 (2.0%)	479 (1.2%)	29 (0.8%)
Other diseases	15,442 (10.5%)	5987 (13.7%)	8657 (23.3%)	12,285 (14.2%)	7324 (18.8%)	913 (24.5%)

Data are presented as *n* (%) unless otherwise indicated. The mean age was reported as the mean ± standard deviation. Training cohort: cohort selected as the training set (before 1 Jan, 2018) to develop the algorithm. Internal testing cohort: cohort used to evaluate the performance of multi-disease diagnosis and radiology abnormality identification (after 1 Jan, 2018). External validation cohort: cohort used to evaluate the model generalizability and robustness in a different center.

*COPD* chronic obstructive pulmonary disease, *ILD* interstitial lung disease, *TB* tuberculosis.

### Ethics and information governance

The current study was performed in compliance with the tenets of the Declaration of Helsinki^26^ and was approved by the Institutional Review Board (IRB)/Ethics Committee of West China Hospital of Sichuan University and Chengdu Shangjin Nanfu Hospital. The requirement for written informed consent was waived because the retrospective data used for system development were de-identified by removing personal information. We applied the updated 30-item Standards for Reporting Diagnostic Accuracy Studies (STARD) 2015 guidelines to our study^27^.

### Evaluation metrics

The DeepMRD^TR^ system aimed to solve multi-label classification problems. A high mean macro area under the receiver operating characteristic curve (AUC) indicated good classification performance. When deployed in real-life scenarios, models that gave a better AUC had a better classification accuracy under the best-chosen cut-off or threshold (operating point). Two-sided *χ*^2^ tests were used to calculate the *p* values and 95% confidence interval (CI) for the differences in accuracy, sensitivity, specificity, and AUC, which were derived using the DeLong technique across a range of classification thresholds. The F1-score metric was used to assess the automated annotation performance of each label, as well as the overall performance of the natural language processing (NLP) model^28^. We also computed the interobserver agreement, which was measured using Cohen’s *κ* statistics, and the system processing time in real-world scenarios to determine whether the system could assist clinicians with diagnosis. Heatmaps generated from gradient-weighted class activation mapping (Grad-CAM)^29^, which were created by gradients flowing into the model’s final convolutional layer before the fully connected layers, were used to evaluate the attentional ability of abnormal regions visually.

### A deep-learning pipeline for the study workflow

**Fig. 1:**
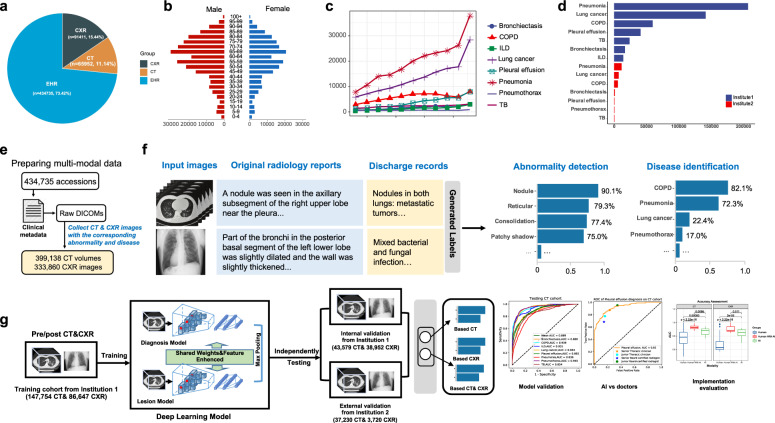
Overview of the framework for developing deep learning models. **a** Initial cohort consists of an EHR dataset and radiograph dataset to develop an NLP algorithm and the deep learning-based algorithm. **b** Patients’ sex and age distribution. **c** The annual occurrence rates of eight major respiratory diseases. **d** Patients’ disease distribution from the two distinct hospitals. **e** Strategy for data collection. **f** NLP model that automatically extracted labels from free-text radiology reports and discharge diagnosis records. **g** Development and validation of a deep learning system to predict 8 major respiratory diseases and 20 radiological abnormalities based on CT/CXR dataset. COPD chronic obstructive pulmonary disease, ILD interstitial lung disease, TB tuberculosis.

The proposed DeepMRD^TR^ system comprehensively simulated the diagnostic thinking of clinical experts. Patient demographics and characteristics of each critical finding from scans are summarized in Fig. 1a–d. The NLP approach for annotating abnormalities and diseases was evaluated in 6274 reports and yielded relatively excellent accuracy (Fig. 1e, f and Supplementary Fig. 2 and Supplementary Tables 1 and 2). For chest abnormalities, the established NLP model achieved an average F1-score of 0.93, a precision of 0.94, and a recall of 0.95. For classifying respiratory disease pathologies, with the discharge diagnosis records as the clinical final decision to make a comparison, the proposed approach achieved an average F1-score of 0.97, with a precision of 0.99, and a recall of 0.94. The above results suggest that the automatically extracted labels were of high quality and could potentially serve as our ground-truth labels for developing DeepMRD^TR^. The DeepMRD^TR^ system consists of four key parts (Fig. 1g and Supplementary Figs. 3 and 4): (1) the CT and CXR standardization module to supply normalized inputs for training and validation; (2) the single-branch chest abnormality identification module; (3) the two-stream major thoracic diseases diagnosis module enhanced by the identification of abnormalities; and (4) the evaluation and visualization module to assess the AI performance and explain the features of the focus region.

### Construction of the DeepMRD^TR^ system

We developed the DeepMRD^TR^ system, which consists of four networks (two CT-Nets and two CXR-Nets) that take either CT or CXR images as input and output for the identification of radiological abnormalities and further prediction of major respiratory disease diagnoses. Simultaneously, we anticipate that the diagnosis results of the AI system can be quantitatively described in the original images using Grad-CAM^29^, alleviating the black box critique of deep neural networks. For the deep-learning-based CT-Nets, two modified 3D-ResNet-18 networks^30^ (Supplementary Fig. 3) were designed for the identification of radiological abnormalities and the diagnosis of major respiratory diseases in 3D CT volumes. The abnormality prediction model generated a total of 20 probability scores, each representing one of the radiological abnormalities investigated. The disease diagnostic model provides a probability score for each of the eight major respiratory diseases based partially on the identified abnormality features. Similarly, the other two modified CXR-Nets based on ResNet-50 (Supplementary Fig. 4) were trained for abnormality description and disease diagnosis on CXR images^31^. To mimic the diagnostic routine of thoracic clinicians, we modified the diagnosis task networks (mentioned above) to design a two-stream disease diagnosis network architecture to perform image feature extraction using the trained backbone of abnormality prediction. Additionally, we fused the extracted feature with another learnable diagnosis pathway using an asymmetric non-local fusion module^32,33^. For both CT/CXR-Nets, we exploited new contrastive learning techniques to improve the efficiency of transfer learning.

### Model performance in identifying multiple abnormalities

**Fig. 2:**
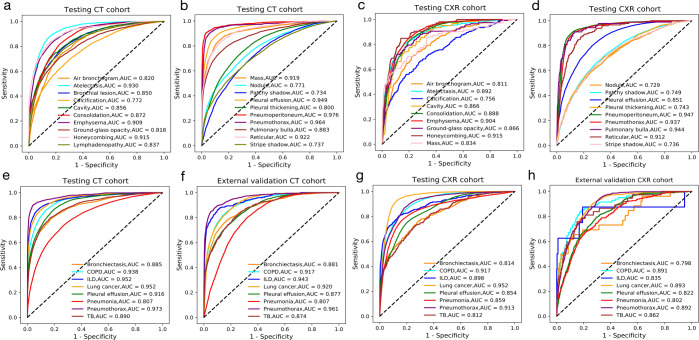
Model performance of the AI system. **a**–**d** ROC curves of AI system in identifying abnormalities based on internal testing CT and CXR cohort. **e**, **f** ROC curves of AI system in making 8-disease diagnoses based on the internal testing and external validation CT cohort. **g**, **h** ROC curves of AI system in making 8 diseases diagnoses based on the internal and external validation CXR cohort. COPD chronic obstructive pulmonary disease, ILD interstitial lung disease, TB tuberculosis.

For the development of the DeepMRD^TR^ system, CT volumes and CXR images from WCH acquired before January 1st, 2018 were assigned to the training set, and those images acquired afterwards were assigned to the internal testing set (4:1 ratio; Supplementary Fig. 1). The CT-Net model could identify 20 chest abnormalities and achieved an average of a multi-way AUC of 0.856 (95% confidence interval (CI):0.843–0.868), with a sensitivity of 0.785 (95%CI:0.764–0.804), and specificity of 0.790 (95%CI:0.785–0.794) for the identification of abnormalities on CT images in our study. The receiver operating characteristic (ROC) curves showed an AUC of 0.930 (95%CI:0.927–0.933) for atelectasis, 0.909 (95%CI:0.906–0.913) for emphysema, 0.919 (95%CI:0.913–0.925) for mass, and 0.976 (95%CI:0.972–0.981) for pneumoperitoneum, and other results at the operating points are shown in Fig. 2a, b and Supplementary Table 3. For the CXR-Net model, bronchial lesion and lymphadenopathy were not identified in CXR images. The CXR-Net model achieved a mean AUC of 0.841 (95%CI:0.832–0.887) in the 18-way classification task, which was lower than that of the CT-Net model. Indicated by the AUC results, the model specialized in abnormalities including 0.904 (95%CI:0.897–0.912) for emphysema, 0.915 (95%CI:0.892–0.932) for honeycombing, 0.947 (95%CI:0.944–0.951) for pneumoperitoneum, and 0.937 (95%CI:0.933–0.942) for pneumothorax. The results in identifying patchy shadow and stripe shadow were less satisfactory, with an AUC of 0.749 (95%CI:0.743–0.755) and 0.736 (95%CI:0.728–0.745), respectively (Fig. 2c, d and Supplementary Table 3).

### Model performance in diagnosing multiple diseases

Two diagnostic classifiers were trained to categorize CT/CXR images into eight common respiratory diseases based on the identification of abnormalities using diagnostic labels automatically generated from discharge diagnosis records. All eight labels for common lung pathologies were derived from real-world clinical reports, and the trained AI system was evaluated on an internal testing dataset. The AI algorithm was performed with an AUC of 0.900 (95%CI:0.872–0.958), a sensitivity of 0.808 (95%CI:0.797–0.821), and a specificity of 0.848 (95%CI:0.845–0.852) for the discrimination of respiratory illnesses on CT images. It achieved an AUC of 0.885 (95%CI:0.878-0.891) for bronchiectasis, 0.938 (95%CI:0.935-0.941) for COPD, 0.952 (95%CI:0.947-0.956) for ILD, 0.952 (95%CI:0.949-0.955) for lung cancer, 0.916 (95%CI:0.914-0.919) for pleural effusion, 0.807 (95%CI:0.803-0.810) for pneumonia, 0.973 (95%CI:0.970-0.978) pneumothorax, 0.890 (95%CI:0.885-0.896) for TB, respectively (Fig. 2e and Supplementary Table 4). For the internal CXR data, the AI system also showed satisfactory performance with an AUC of 0.866 (95%CI:0.832–0.887), sensitivity of 0.805 (95%CI:0.785–0.824), and a specificity of 0.786 (95%CI:0.783–0.790) for the overall classification of chest respiratory diseases. It achieved an AUC of 0.814 (95%CI:0.797-0.835) for bronchiectasis, 0.917 (95%CI:0.913-0.921) for COPD, 0.898 (95%CI:0.882-0.913) for ILD, 0.952 (95%CI:0.950-0.953) for lung cancer, 0.854 (95%CI:0.851-0.857) for pleural effusion, 0.859 (95%CI:0.855-0.863) for pneumonia, 0.913 (95%CI:0.908-0.917) pneumothorax, 0.812 (95%CI:0.790-0.830) for TB, respectively (Fig. 2g and Supplementary Table 4).

### Robustness of the AI system in various conditions

As the trained deep learning model could be deployed in different hospitals where the population, scanning conditions, and patient disease severity may differ from those in the training data, the AI system was also evaluated in terms of its robustness in a different hospital (CSJH) with different resource levels, screening machines, and scanning periods (Table 1 and Supplementary Fig. 1). The data processing procedures were consistent with those used in the training and internal testing cohorts. For the external CT cohort (Fig. 2f and Supplementary Table 5), the AI system achieved a mean AUC of 0.882 (95%CI:0.825-0.908), a sensitivity of 0.807 (95%CI:0.786-0.826) and a specificity of 0.804 (95%CI:0.800-0.807) in the diagnosis of major respiratory diseases. With regard to the external CXR cohort (Fig. 2h and Supplementary Table 5), the AI system demonstrated a mean AUC of 0.841 (95%CI:0.801-0.884), a sensitivity of 0.811 (95%CI:0.733-0.869) and a specificity of 0.761 (95%CI:0.748-0.733) in the discrimination of major thoracic diseases based on chest radiographs. The multi-label abnormality results of the external cohort are provided in Supplementary Fig. 5. With relatively poor image quality from another hospital, the model still has relatively good performance, suggesting that the model can still obtain a stably favorable result and can be applied in resource-restrained health settings.

Moreover, it is common for deep-learning or machine-learning-based models to perform relatively worse on unseen datasets owing to differences in data distribution and possible overfitting on the training data. Normally, such a problem can be alleviated by collecting more data or training deep models using data augmentation. As demonstrated in Supplementary Fig. 6, models trained with full-scale data outperformed those trained with part of the data, exhibiting improved generalization ability. With more training data, the performance improved steadily until it was saturated at 80% of the full-scale dataset.

### Relative performance on CT and CXR images

**Fig. 3:**
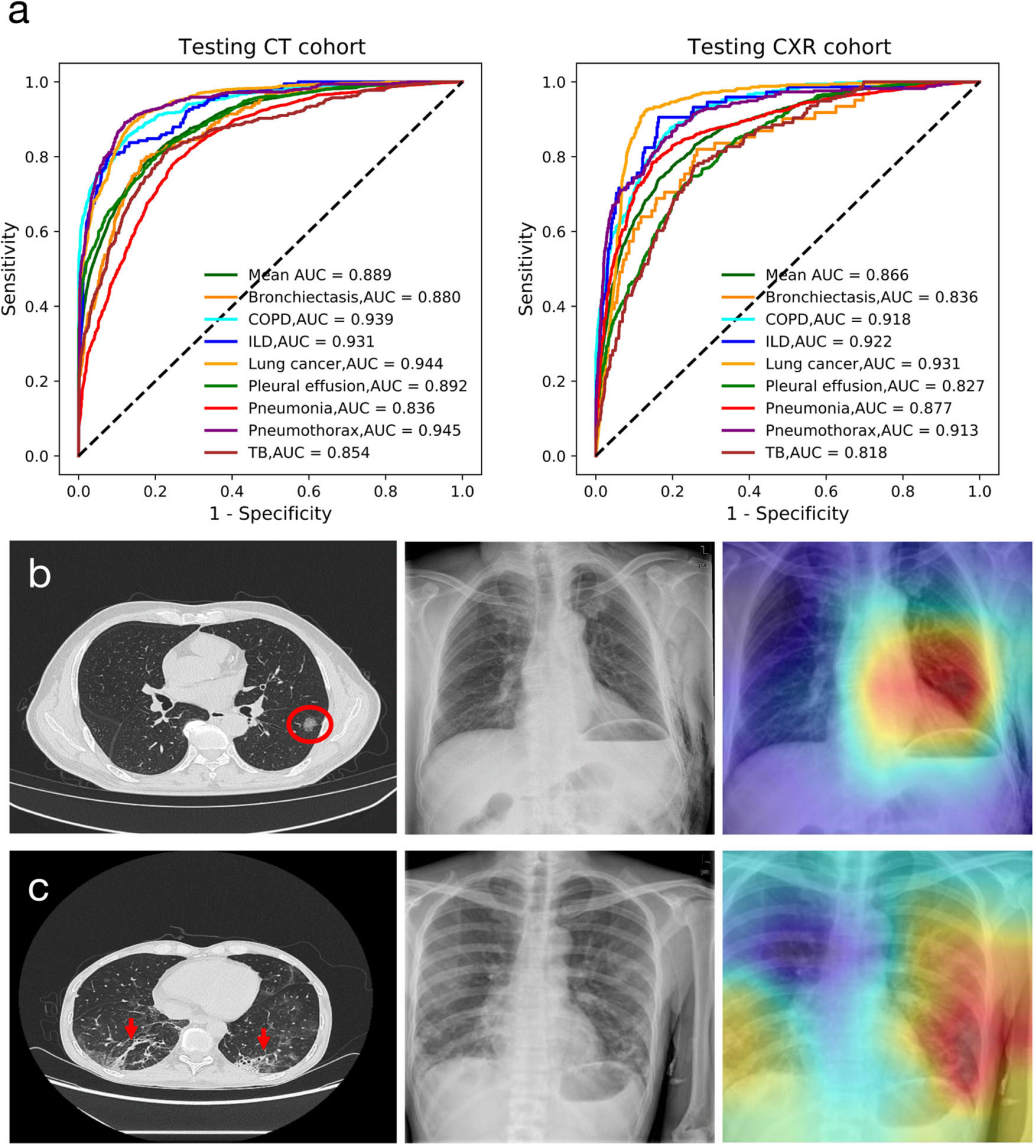
The relative performance of AI system for the CT and CXR cohorts of the same patients for multi-disease diagnosis. **a** ROC curves of AI system in making diagnoses of the included eight diseases based on the CT and CXR cohorts. **b** Patient with lung cancer who had a proper diagnosis by all readers on the CT scan (red circle) but incorrect predictions on the CXR, whereas the AI system could precisely localize the lesion location. **c** A case with pneumonia where all readers correctly identified the infectious lesions (red arrows) on the CT scan but made wrong diagnoses based on the CXR images. COPD chronic obstructive pulmonary disease, ILD interstitial lung disease, TB tuberculosis.

To better understand the relative efficacy of CT and CXR images in diagnosing major respiratory diseases, we devised both CT-based and CXR-based techniques and tested them using previously unseen paired data (same patients with both CT and CXR examination during the same time period). By comparing the relative performance of the CT-based AI system and CXR-based AI system, we can determine the diseases on which the diagnostic accuracy of human + AI using CXR images can reach that of human alone using CT images, and get clues on which diseases are not suitable for CXR screening even in the presence of an AI assistant. In this paired cohort, the ROC curve (Fig. 3a) showed that the macro-mean AUC of the eight categories was 0.889 for the CT cohort and 0.866 for the CXR cohort. In practice, clinical experts are also asked to make diagnoses on the CT & CXR cohorts with and without the assistance of the AI system. It turns out clinical experts alone observe fewer lesion regions on CXR images than with the assistance of the AI system. Figure 3b, c shows two examples of senior readers as well as the AI-corrected diagnosis on CT and CXR images. Most readers initially were not able to detect lesions on the CXR images that were precisely diagnosed by the AI system on both CT and CXR images. With the assistance of the AI system, those lesions could be correctly identified on the CXR images by most tested readers.

### Comparison between the DeepMRD^TR^ system and practicing radiologists and thoracic clinicians

Consistent with previous studies, we compared the AI system and licenced medical workers in the same cohort regarding the abnormality detection task and disease diagnosis task. Eight clinical specialists from WCH or CSJH with a wide variety of expertise-junior readers (with less than 7 years of clinical experience) and senior readers (with more than 7 years of clinical experience). They were enrolled from different departments (respiratory and radiology) and were blinded to the case review. We compared the performance of DeepMRD^TR^ to that of human readers from electronic health records (EHRs) discharge diagnosis records, extracted labels from which are defined as the golden standard.

**Fig. 4:**
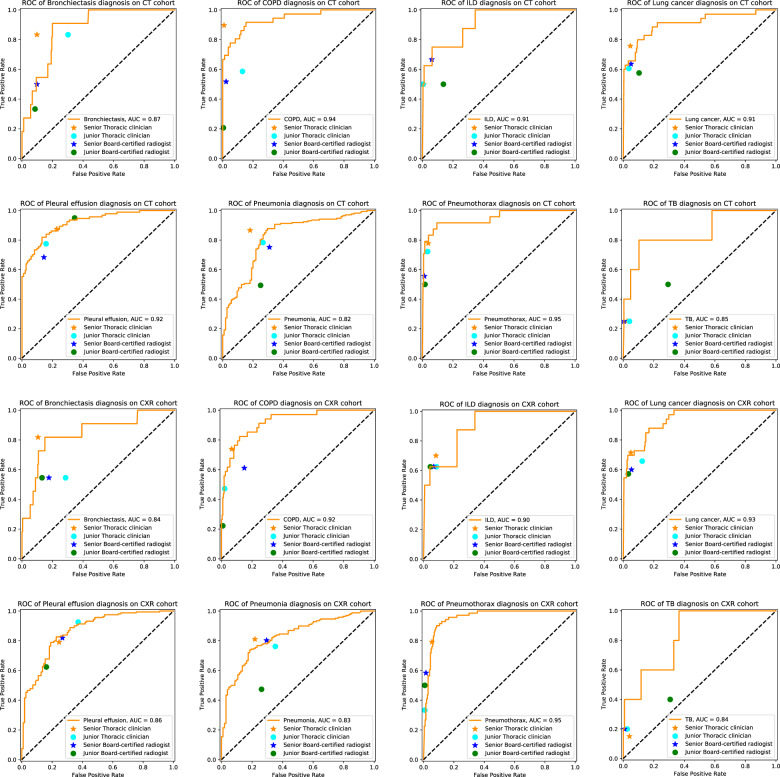
Model performance of the AI system in making multi-disease classification compared with experts on the CT and CXR cohorts. ROC curves for diagnostic performance in the comparison between our AI system and four groups of experts (senior/junior thoracic clinicians and senior/junior radiologists). COPD chronic obstructive pulmonary disease, ILD interstitial lung disease, TB tuberculosis.

In a reader study involving eight experts and CT images, the AI system achieved a performance equivalent to that of senior human experts in the diagnosis of interstitial lung disease (ILD; AUC = 0.91) and pleural effusion (AUC = 0.92). Senior thoracic clinicians were better than the AI system in identifying bronchiectasis, chronic obstructive pulmonary disease (COPD), lung cancer, and pneumonia. For pneumothorax and TB, the AI outperformed the readers, demonstrating AUCs of 0.95 and 0.85, respectively. For human-DeepMRD^TR^ comparison based on the CXR cohort, the AI system yielded a similar performance to senior experts in COPD, lung cancer, pleural effusion, and pneumothorax diagnosis, with equivalent accuracy. The performance of the AI system for bronchiectasis, ILD, and pneumonia diagnosis was inferior to that of senior clinicians but superior to or on par with that of junior experts. In consistent with the CT cohort, AI also outperformed the readers for TB diagnosis in the CXR cohort (Fig. 4).

### Combining diagnoses from the DeepMRD^TR^ system and experts

To verify the feasibility of incorporating our AI system into real-life clinical workflows in a human + AI fashion, we evaluated whether the system could assist respiratory clinicians and radiologists with their diagnoses while attempting to quantify the improvement. First, we employed an assessment approach to investigate the performance beyond AUC to establish the threshold selection by considering the trade-off between sensitivity and specificity to match different expert groups. Second, to assess the deep learning system for clinical implementation, we compared the time required to generate a clinically acceptable diagnosis, with and without the assistance of the DeepMRD^TR^ system.

We calculated the sensitivity and specificity of readers’ eight different binary classifications, as well as the AI system’s threshold score, to match readers’ sensitivity and specificity in Table 2. For example, at the same sensitivity, the DeepMRD^TR^ system performed better in terms of specificity than the junior radiologists (0.929 vs. 0.880). We also explored the potential involvement of the system in increasing the diagnostic performance of senior/junior clinicians and senior/junior radiologists in the workflow. When using the majority vote and weighted error over the predicted classes of multiple images for each patient, the combined result achieved a sensitivity of 0.673 (95%CI:0.652–0.694) and specificity of 0.912 (95%CI:0.890–0.918) for junior radiologists, achieving a significant improvement compared to that with the sensitivity of 0.569 (95%CI:0.546–0.579) and specificity of 0.880 (95%CI:0.870–0.894) without the assistance of the AI system (Table 2).

**Table 2:** Comparison and combination of sensitivity and specificity between experts’ reading results and the proposed DeepMRD^TR^ system.

	Experts		DeepMRD^TR^ System		Experts + AI	
	Sensitivity	Specificity	Sensitivity	Specificity	Sensitivity	Specificity
Junior radiologists	0.569(0.546–0.579)	0.880(0.870–0.894)	0.570(0.527–0.608)	0.929(0.917–0.943)	0.673(0.652–0.694)	0.912(0.890–0.918)
Junior clinicians	0.617(0.556–0.678)	0.878(0.847–0.902)	0.618(0.578–0.654)	0.922(0.905–0.936)	0.665(0.642–0.696)	0.916(0.912–0.937)
Senior radiologists	0.608(0.582–0.616)	0.929(0.928–0.952)	0.608(0.568–0.643)	0.933(0.923–0.937)	0.683(0.675–0.688)	0.954(0.948–0.957)
Senior clinicians	0.748(0.732–0.759)	0.942(0.920–0.968)	0.750(0.713–0.788)	0.898(0.882–0.914)	0.762(0.759–0.781)	0.953(0.950–0.961)

**Fig. 5:**
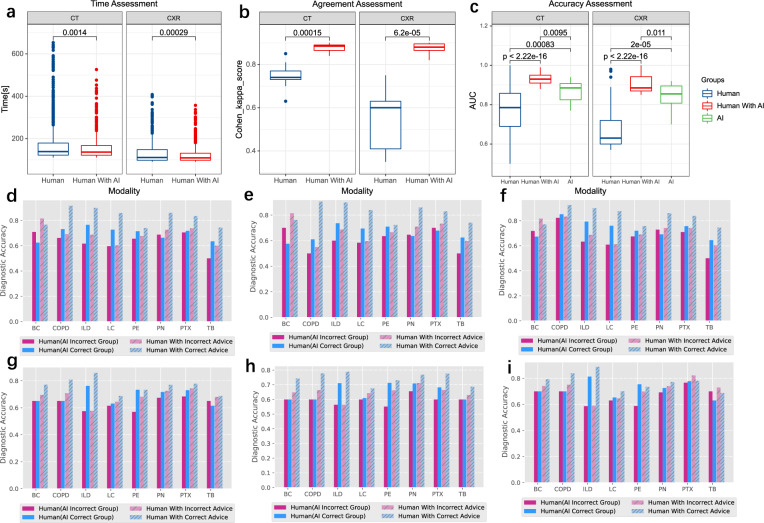
Comparison of human-only, human with AI, and AI-only diagnoses for clinical radiation implementation. **a** Time assessment of diagnosis without and with the assistance of the deep learning system. **b** Agreement of eight experts in disease diagnosis without and with the assistance of the deep learning system **c** displays the performance of the AI approach (AI only), the current clinical workflow (human only), and the AI-assisted approach (human with AI). Each box represents the interquartile range (IQR, 25th and 75th percentiles) and the center line represents the median of the results. The whiskers represent minimum and maximum data points, excluding outliers. Outliers are defined as greater than the 75th percentile +1.5 × IQR and smaller than the 25th percentile −1.5 × IQR and are denoted as nodes. **d** Mean diagnosis performance among a diverse range of human readers with correct/incorrect AI advice based on CT images. **e**, **f** Diagnosis performance of junior and senior readers with various AI advice based on CT images. **g** Mean diagnosis performance among a diverse range of human readers with correct/incorrect AI advice based on CXR images. **h**, **i** Diagnosis performance of junior and senior readers with various AI advice based on CXR images. BC bronchiectasis, COPD chronic obstructive pulmonary disease, ILD interstitial lung disease, LC lung cancer, PE pleural effusion, PN pneumonia, PTX pneumothorax, TB tuberculosis.

In terms of implementation in clinical workflow with the aid of an AI assistant, the amount of time that human doctors spent on making a diagnosis decreased slightly (145 s [interquartile range (IQR), 129–182] vs. 144 s [IQR 128–175] for CT images; *p* = 0.0014; and 104 s [IQR 99–150] vs. 103 s [IQR 99–143] for CXR images; *p* < 0.001) compared to the original clinical workflow. Simultaneously, the mean agreement (Cohen’s *K*) among the eight doctors performing diagnosis increased significantly from a median of 0.746 without AI assistance to 0.877 with AI assistance for CT images, and from 0.600 to 0.865 for CXR images (*p* < 0.001). Furthermore, no significant performance differences were found among the AI approach (AUC = 0.890), original clinical workflow, and AI-assisted approach (*p* < 0.001) (Fig. 5a–c).

For the failure analysis of incorrectly classified cases, we also examined the diagnostic performance of human readers with correct and incorrect AI advice in each case. As illustrated in Fig. 5d–f, AI pre-diagnosis advice based on CT images, whether predicted correctly or incorrectly, would assist the doctor’s diagnosis to a certain degree. Even when AI-based classification is incorrect, there is a high possibility that abnormalities would be found in the lesion area by doctors, which indicates that AI, to some extent, provides doctors with subtle indicators. In contrast, as shown in Fig. 5g–i, for CXR images, the different types of AI advice had large gaps in diagnostic accuracy. In addition, AI advice was more advanced for junior doctors than senior doctors.

### Interpreting the DeepMRD^TR^ systems

In the proposed system, to show some representative subjects for visualization, we explored the channel’s attention in this network to determine which channel map provides discriminating information, how pathological abnormalities arise in the spatial dimension, and which scale is an important aspect of the diagnostic. As a commonly used method for interpreting this AI diagnosis black box, the class activation map (CAM) may provide participative focus regions for each unique prediction from the model, which is connected to the back end of the diagnostic model. The results showed that our system extracted powerful features to distinguish between different categories in the latent space (Supplementary Fig. 7). When CAM covers a broad range or provides partial coverage of diagnostic areas used by human experts, it can improve doctors’ sensitivity and confidence in their diagnosis.

## Discussion

In this study, we proposed an effective deep-learning-based medical image interpretation system, which was trained on a large-scale real-world dataset of CT/CXR images with automated annotations extracted from free-text reports and discharge diagnosis records via NLP techniques. Our deep learning algorithms achieved promising accuracy in identifying about 20 types of radiological abnormalities and further classified eight common respiratory diseases. For external validation, our model also yielded perfect classification performance, demonstrating the generalizability and applicability of the AI system under a limited domain shift. Simultaneously, the comparison between performances of human and DeepMRD^TR^ indicated that junior clinicians might reap more benefits or more substantial improvement than senior clinicians from this system. Finally, the preliminary version system was implemented within the workflow to estimate the ability to contextualize to the clinical context (Supplementary Fig. 8). Such an AI system may be feasible to automate the triage process by prioritizing scans with suspicious abnormalities requiring earlier human assessment, which could shorten the turnaround time of multidisciplinary diagnostic workflow, decrease the waiting time of patients, lessen clinicians’ workload, and allow these doctors to respond more effectively in the diagnosis of respiratory diseases.

The current study is innovative and distinguishable from other studies in the field in the following respects. Generally, the greater the amount of data, the higher generalization and robustness of the model obtains. In this work, a heterogeneous quantity of realistic datasets was collected to train, optimize and validate the DeepMRD^TR^ system, which was possibly the largest in the field of abnormality description and thoracic disease identification compared with previous studies, including the massive sample size of 1,294,475 EHRs from 434,735 real-world patients, 228,563 CT volumes, and 129,319 CXR images, discharge diagnosis-derived high-quality and reliable ground truth labels, were collected to train, optimize, and validate the DeepMRD^TR^ system, which is larger than the reported sample size in several previous studies^8,21,34–38^. Second, on account of the multiple co-existent diseases and imaging abnormalities, both of which were essentially different. The DeepMRD^TR^ system could strictly distinguish abnormalities from diseases, simultaneously localize the majority of chest abnormalities (*n* = 20) and further identify eight major thoracic diseases. Remarkably, the classifier for bronchial lesions was firstly reported in our study. This study disentangled abnormality findings and thoracic diseases into two separate models (this is the first attempt, to the best of our knowledge), and could be more readily aligned to clinical deployment. In real-life clinical scenarios, a patient might harbor more than one major illness; thus, our model attempted to account for common respiratory diseases rather than one specific disorder. Previous studies have focused on only one disease^22–24,37^ or adopted a mixture of abnormality description and disease diagnosis labels, which could not be properly deployed in clinical practice. For example, the famous CheXpert and CheXNeXt^36,37^ include 14 classes such as consolidation, lung opacity, and pneumonia. The former two classes are descriptions of chest abnormalities, whereas pneumonia belongs to the classification of thoracic diseases. However, it is confusing to include them in a single model for prediction and implementation. Third, aside from the advanced 3D ResNet architecture and contrastive learning techniques, the final thorax disease prediction model comprehensively simulated the diagnostic process of human experts by incorporating abnormality description features into the prediction pipeline. The DeepMRD^TR^ system comprises three models, including the NLP, CT-Net, and CXR-Net models, and is designed to provide the final disease prediction as well as quantitative possibilities of lesion characteristics. In comparison with other work, the CAM heatmaps were provided to enhance model interpretability which augmented the clinical utility^37,39^.

We developed and validated the DeepMRD^TR^ system with the aim to streamline the CT/CXR scan interpretation workflow. Our model makes a diagnosis by simulating the reasoning process of an expert clinical worker. The AI-assisted workflow in real-world practice starts with installing “DeepMRD^TR^” offline in the hospitals. When a patient undergoes a radiological examination, our AI system will automatically take CT volumes or CXR images as input, subsequently, process the data, then analyze the suspicious regions of interest, generate the CAMs, and output final abnormality predictions and disease diagnosis nearly instantaneously. There might be several challenges including incompatibility of such systems with local medical equipment, and additional patient waiting time due to model inference to achieve an “actionable” diagnosis, thus limiting the actual clinical deployment. With regard to these issues, we have developed easy-to-obtain docking between the DeepMRD^TR^ system and radiology picture archiving and communication system (PACS) or image scanners to make the software available. Thus, the time to achieve an “actionable” diagnosis by our system is negligible and the overall diagnosis time could even be shortened, leading to optimization of the established clinical workflow through the integration of DeepMRD^TR^ (Fig. 5).

The human-DeepMRD^TR^ comparison revealed more obvious performance improvements in early career physicians relative to senior clinicians, indicating that our system could conduce to upgrade the interpretation quality. Moreover, our model could assist a rapidly increasing number of experts grown from less-experienced clinicians, providing reliable advice without the limits of time and space. This model could be generalized in diverse clinical scenarios. For instance, in our other work, we employed vehicle-mounted CT devices installed with a deep algorithm to screen lung cancer^40^. Similarly, the “DeepMRD^TR^” system will be installed in these mobile CT devices and then rapid triage will be provided in remote areas, where either experts or high-tech facilities are scarce.

We developed the DeepMRD^TR^ system to complement the current clinical workflow, rather than subvert it, and to assist human physicians, as opposed to replacing them. The clinical value of AI systems might signify that, in the context of mounting complicated cases, clinical workload, and medical documents, healthcare workers could harness the best of AI to enable gains in operational efficiency, and meanwhile the AI model could achieve higher diagnostic accuracy and robustness via active learning where feedback from physicians will be furnished to AI algorithms in the form of increasing training data^41^. In the future, our model would be expected to have crucial implications in clinical community settings, alerting and containing early respiratory diseases (i.e., COVID-19, or SARS), or longitudinally monitoring individuals during the course of treatment to evaluate the efficacy of interventions in the elementary healthcare institutions. Further studies are warranted to determine the optimal workflow and implementation of AI-based algorithms in healthcare settings.

However, some limitations merit consideration in our study, hopefully, which we can resolve in the future. Given that the AUC has been considered as a relatively effective performance metric for disease prediction in academic research^42^, the AUC was utilized to evaluate our deep-learning model and to compare human/DeepMRD^TR^ performances. While the AUC alone has limited practical utility, notably, it is still ongoing to select the proper operating points adopted in clinical workflow, taking account of outcomes and cost^43^. Second, the annotation biases introduced by the large-scale image dataset could affect the performance of abnormality detection and disease diagnosis and should be taken with caution. The labels of the training images were text mined from EHRs utilizing NLP, and a comparison of NLP labels vs. manual ground-truth annotations would be intriguing but unrealistic, owing to the inaccessibility of annotations from clinical experts for such a large training set^44^. Third, our patients were all Asians, which could potentially limit the generalizability of our AI system to other international regions. Additional validation across populations from American and European hospitals is warranted to further validate the reported performance^45^. Fourth, selection biases were resulted from choosing a subset of radiological abnormalities for prediction would lead to selection bias. Finally, the number of participating clinicians, coupled with retrospective data vs. prospective validation, limited the actionability of the report. In the foreseeable future, the increased use of DeepMRD^TR^ will empower clinicians in routine clinical workflows.

Finally, this study demonstrated the value of an AI system in distinguishing between a wide range of chest abnormalities and various thoracic diseases using a deep learning platform with a comparison against senior/junior doctors’ performance on a large-scale dataset, offering clinical experts the potential of a fast versatile triage tool that leverages deep learning to improve operational efficiency and ultimately enhance clinical decision-making. Future well-designed prospective studies and algorithm performance improvements will expand its application and feasibility for the diagnostic assessment of all lung disorders.

## Methods

### Patient cohort and data collection

This study retrospectively collected CT/CXR data with accompanying EHRs from inpatients enrolled between October 2008 and February 2021. We selected eight common respiratory diseases, including bronchiectasis, COPD, ILD, lung cancer, pleural effusion, pneumonia, pneumothorax, and TB according to the International Guidelines for Diagnosis and Treatment of Respiratory Diseases based on Murray and Nadel’s textbook of respiratory Medicine^46^. We covered 20 radiological abnormalities, including air bronchogram, atelectasis, bronchial lesion, calcification, cavity, consolidation, emphysema, ground-glass opacity, honeycombing, lymphadenopathy, mass, nodule, patchy shadow, pleural effusion, pleural thickening, pneumoperitoneum, pneumothorax, pulmonary bulla, reticular, and stripe shadow. The following inclusion criteria were used to screen patients’ eligibility: (1) hospitalized inpatients diagnosed with major respiratory diseases; (2) inspected with thoracic CT or CXR scans; and (3) had access to EHRs, including at least discharge diagnosis records or radiology reports. After screening, patients were further excluded based on the following criteria: (1) having only postoperative images; (2) being diagnosed with rare diseases other than the eight major respiratory diseases we defined; (3) being under the age of 18; (4) radiological studies with image reconstruction kernels unrelated to the lung and view positions unrelated to the chest (e.g., only AP/PA were reserved), or having views with motion artifacts.

For the EHR data collection in our study, ideally, for a unique patient, his/her EHR data should include at least two basic pieces of information, that is, radiology reports and discharge diagnosis reports in line with international standards (for example, ICD-10). Other information, such as basic condition, disease course, prescription, and medical examination documents issued by the doctor, could also be used. Sensitive information contained in these EHR data should be desensitized in accordance with the relevant requirements, regulations, and standards of the state and competent departments of the medical and health industry for the protection of user privacy data in these fields.

### Ground-truth labels

In this study, radiological reports and multi-modal discharge diagnosis records were used as the gold standards for abnormality detection and disease diagnosis. To train the model, given that manually annotating the classification of abnormalities/diseases according to the original records can be too time-consuming, it is necessary to leverage automated label extraction techniques to create a large-scale labeled dataset containing CT/CXR data and linked abnormality/disease labels using the NLP method. On the validation dataset, for a fair comparison, the performance of the DeepMRD^TR^ was compared to that of human readers using reviewed diagnosis records from EHR as the gold standard.

During the development of these automated label extraction models, a modest quantity of training data (*n* = 1000) was manually annotated by a group of medical specialists. Patients were requested to mark the presence or absence of abnormalities and diseases according to the original radiology reports and discharge diagnosis records, respectively. At least two human experts were involved in the annotation of each free text report. Annotation results were compared to reach a consensus. In the event of inconsistent annotations, an extra human expert was introduced to make the final arbitration.

### Radiology data standardization

We collected a radiology dataset using two modalities (CT and CXR). Both CT and CXR data were collected by selecting scans obtained at hospitals and dated from Oct, 2008. This study only included CT and CXR data together with relevant EHRs. To create abnormality and disease labels, the built NLP system was used to automatically assess the related radiological reports and discharge diagnosis records. The CT images were standardized to 64 × 256 × 256 to preserve as much detail as feasible in the axial axis while reducing the computational expense. Furthermore, all CXR scans were collected at a resolution greater than 886 × 886 pixels and subsequently normalized to 1024 × 1024 pixels. Other data pre-processing methods also included data denoising, enhancement, and rotation to increase the robustness of the network.

### NLP model development

The description of the NLP model is shown in Supplementary Fig. 2. In particular, the models took free-text radiology reports or discharge diagnosis records as inputs and output a set of discrete binary labels for multiple abnormalities and diseases, respectively. Patient records or reports vary significantly in length and density of data points; therefore, we vectorised the data into a form with multiple lines, each with a specified length of 200, to facilitate further processing. If the sentence length is less than the specified value, special symbols will be automatically filled at the end by default. If the sentence length is greater than the specified value, the first 200 will be retained by default, and the redundant part will be truncated. Each comment becomes a uniform-length index vector after data vectorization, and each index corresponds to a word vector. The text classifier, which can be used for automatic label creation, was created using supervised learning. Specifically, we fine-tuned a CNN-based text classifier on the aforementioned labeled text-label pairs, whose text features were extracted by BERT^47^.

### CT-Net framework

Based on the learning targets, we developed two CT-Nets for abnormality description and disease diagnosis. These two models followed distinct model designs and were separately trained using different image/label pairs. To efficiently extract representative features from the 3D volumetric input, we used a modified ResNet-3D-18 backbone as a feature extractor. The obtained features are combined with the input of the residual module as the final output of the residual module. In particular, unlike the vanilla architecture, we neglected the first *z*-axis pooling operation to increase the resolution of the final feature maps along the *z*-axis. A multiple binary cross-entropy loss function was employed to supervise the multi-label classification task.

Clinical experts first reviewed the CT volume for abnormal findings before making a decision based on comprehensive reasoning about the results. Inspired by this clinical routine, we created a two-stream architecture for disease diagnosis that used the previously trained abnormality description model for efficient feature representation of abnormal CT volume data (Supplementary Fig. 3). Specifically, this architecture uses an asymmetric nonlocal fusion module to fuse abnormal features with a learnable diagnosis route. To achieve advanced transfer learning efficiency, we adopted a variable-dimension transform-based method to pre-train the 3D ResNet, whose parameters were used to initialize the abnormality and diagnosis backbone^48^.

### CXR-Net framework

The CXR architecture design follows the concept described in the aforementioned section for CT-Nets, where the abnormality model was a single-pathway network and the diagnosis model adopted a dual-pathway structure. We used a ResNet-50 backbone with 2D convolution blocks instead of ResNet-3D-18 for feature extraction from the 2D CXR images. We developed a mix-up-based contrastive learning strategy to pre-train ResNet-50 utilized in CXR-Nets to assist effective transfer learning from in-domain representations. Supplementary Fig. 4 depicts the contrastive learning process in greater detail. The contrastive learning model learns advanced transferrable CXR image representations from unannotated images in an unsupervised manner. Specifically, it learns to distinguish instances in a momentum-updating framework. As illustrated in the “Feature Encoding” parts, parameters in the green network (bottom) were updated using gradient backpropagation, while that in the gray network (upper) were updated with momentum update as follows: ${\rm{net}}_{{\rm{grey}}} = \theta \ast {\rm{net}}_{{\rm{grey}}} + \left( {1 - \theta } \right) \ast {\rm{net}}_{{\rm{green}}}.(f_1^o,f_2^o),(f_1^m,f_2^m),(f_m,f_2^m)$ are positive feature pairs that must be pulled closer to each other in the feature space. For training, we employed an info Noise-Contrastive Estimation loss adapted for the momentum update architecture, which drove the model to separate different image instances and group similar images using data augmentation or image and feature level mix-up. In this study, the contrastive learning model was trained using large-scale public CXR datasets such as ChestX-ray14^34^, CheXpert^36^, MIMIC-CXR^49^, and MURA^50^ to obtain a pre-trained backbone network, which was fine-tuned from the learned pre-trained parameters using transfer learning to obtain advanced prediction performances.

### Network training strategy

For training and testing, we used the PyTorch^51^ deep-learning framework on 8 × NVIDIA TITAN RTX GPUs. The Adam optimizer^41^ with a weight decay of 0.0001 was used to train the CT-Nets. The initial learning rate was set at 0.0005, and the learning rate decayed by a factor of 10 after the 35th, 40th, and 43rd epochs. All models were trained for 45 epochs. Owing to the restricted GPU memory, the batch sizes on each GPU were set to 16 for the abnormality model and 8 for the disease model.

To train CXR-Nets, an Adam optimizer with a weight decay of 0.0001 was used. The initial learning rate was set at 0.0005, and the learning rate decayed by a factor of ten after the 25th and 35th epochs. All models were trained for 45 epochs. Owing to the restricted GPU capacity, the batch sizes on each GPU were adjusted to 128 for the abnormality model and 64 for the disease model.

### Reporting summary

Further information on research design is available in the Nature Research Reporting Summary linked to this article.

## Supplementary information

Supplementary Materials

Reporting Summary

## Data Availability

The main data supporting our results in this study are almost all available in the manuscript and Supplementary Information. We are sorry that the raw data from hospitals cannot be made publicly available because of hospital regulation restrictions and privacy concerns to protect our patients. Anonymized data might be accessible for research purposes from the corresponding authors upon reasonable request.

## Code availability

The codes that support the findings of this study are available as follows: https://github.com/chiehchiu/DeepMRD.
