# Supplementary material for: Development and validation of an abnormality-derived deep-learning diagnostic system for major respiratory diseases
Source: NPJ Digit Med. 2022 Aug 23;5:124. doi: 10.1038/s41746-022-00648-z (PMC9395860; doi:10.1038/s41746-022-00648-z)
Supplement: Supplementary file 1 — Supplementary Materials [file 41746_2022_648_MOESM1_ESM.docx]

**Supplementary materials**

**Supplementary Figures**

**
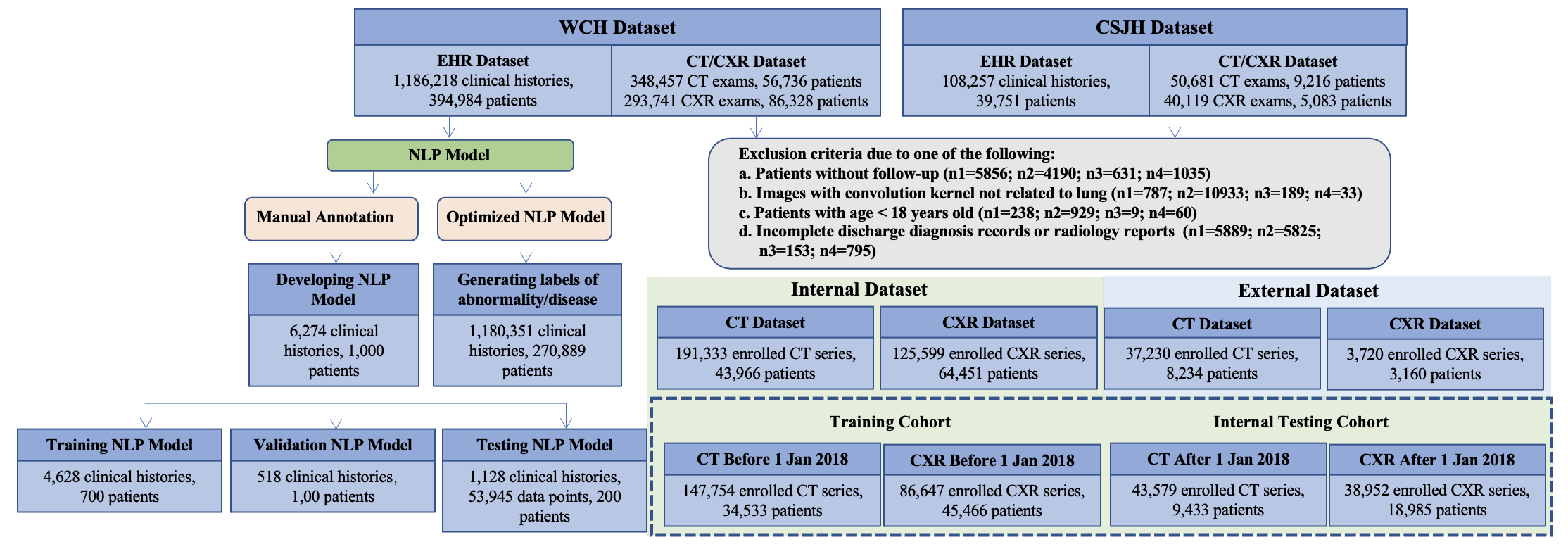
**

**Supplementary Figure 1. Data flow diagram showing the inclusion and exclusion of EHR and CT/CXR image datasets.** Using WCH data collected between October 2008 and January 2020, we developed an NLP algorithm and a deep learning-based algorithm utilizing an EHR dataset and CT/CXR image datasets. To validate the model performance, an independent external validation cohort (CSJH) was collected between April 2012 and February 2021, where n1 and n2 represented the exclusion of CT and CXR images in WCH cohort, respectively, and n3 and n4 represented the exclusion of CT and CXR images in CSJH cohort, respectively. The data split and the corresponding usage of the internal and external cohort were distributed as follows: (**a**) training cohort: the CT cohort (147,754; n=34,533) and the CXR cohort (86,647; n=45,466) were picked as the training set for developing the DeepMRD^TR^ system (before 1 Jan 2018); (**b**) internal testing cohort: the CT cohort (43,579; n=9,433) and the CXR cohort (38,952; n=18,985) were used to evaluate the performance of proposed DeepMRD^TR^ system for abnormality detection and disease diagnosis (after 1 Jan 2018); (**c**) external validation cohort: the CT cohort (37,230; n=8,234) and the CXR cohort (3,720; n=3,160) were used to evaluate model robustness in another institution.

CSJH Chengdu ShangJin Nanfu Hospital, CT Computed tomography, CXR chest X-ray, EHR electronic health record, NLP natural language processing, WCH West China Hospital.


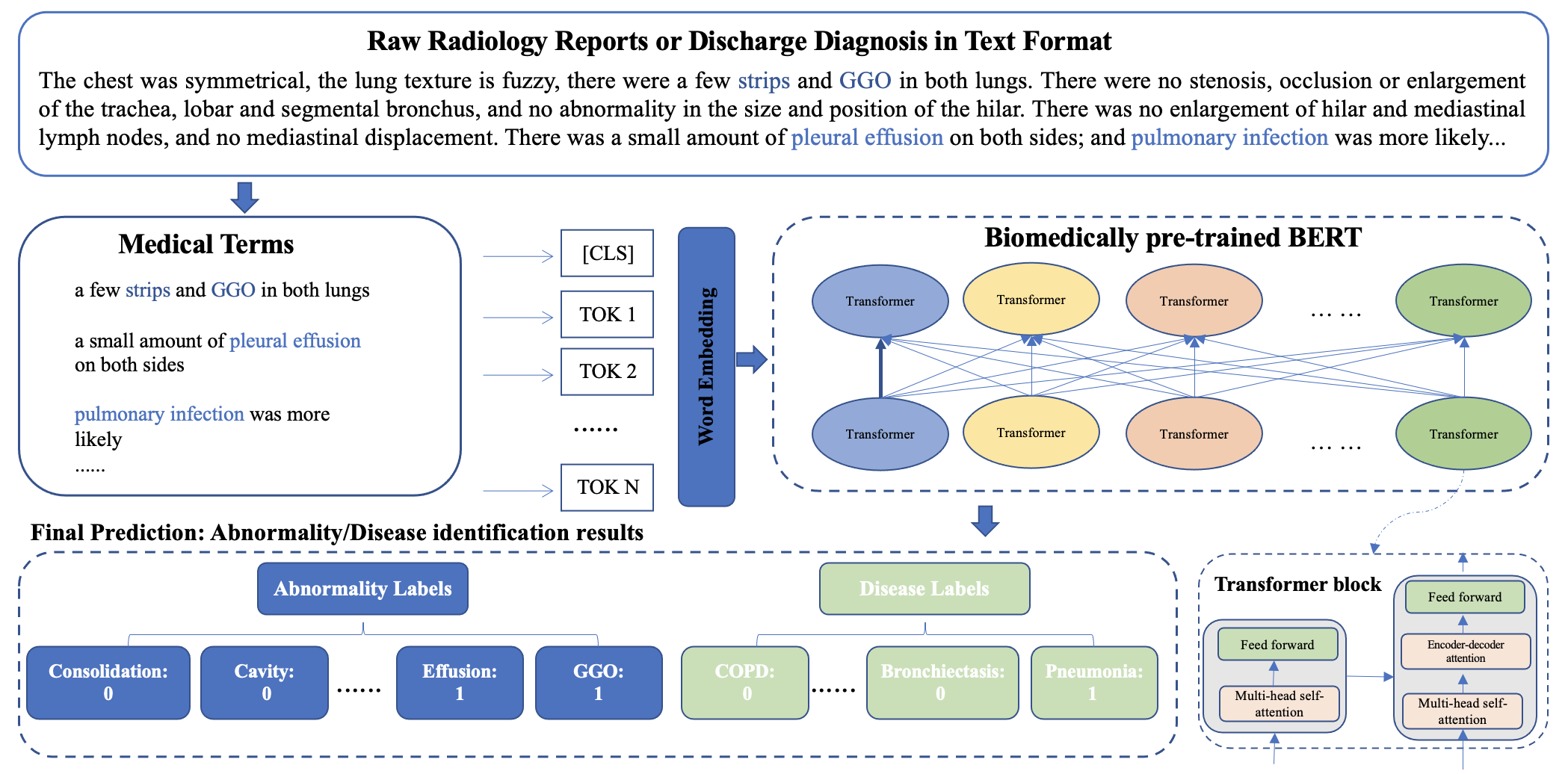


**Supplementary Figure 2. Automatic label extraction module.** Based on the free text of radiology reports and discharge diagnostic records, the NLP algorithm was built to predict patients' radiological abnormalities and discharge diagnoses in a structured label format (shown in the lower left part). To construct uniform length index vectors, the raw-texts were initially fed into a data vectorization procedure. The text-classifier was then trained using supervised learning on a modified BERT, which may be utilized for automatic label generation. The pre-annotated text-label pairings were used to train the text-classifier. The model was used to extract labels of the radiological abnormalities and discharge diagnoses on a total of 1,180,351 clinical history reports from 270,889 patients. NLP natural language processing.


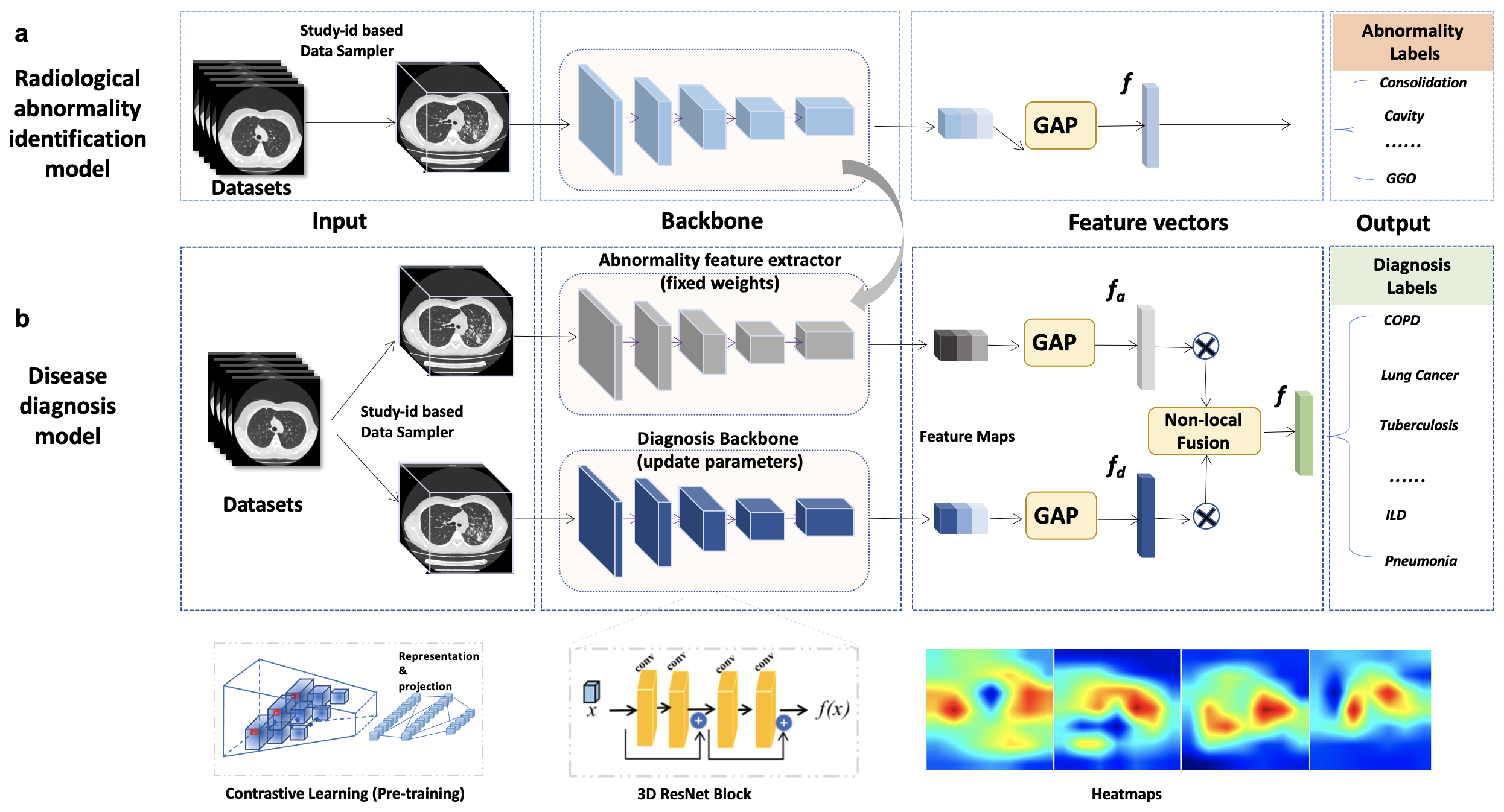


**Supplementary Figure 3. CT-Net framework. a** The radiological abnormality description model was developed by applying a 3D ResNet-18 feature extractor to each 3D volume and employing multiple binary cross-entropy loss function, each for one target of abnormality class. **b** The second row illustrated the model architecture for the disease diagnosis model. It’s a dual-pathway architecture that adopted the trained abnormality prediction backbone for abnormality feature extraction and fused them with a learnable diagnosis pathway using an asymmetric non-local fusion module. The parameters of the abnormality model’s backbone were directly transferred from previously trained networks and were kept fixed during diagnosis model training. It should be noted that the CXR-Nets built on a similar architecture concept, with the 3D ResNet-18 backbone being replaced with ResNet-50.

**Supplementary Figure 4. Contrastive learning framework used for training the CXR-Net.** To facilitate efficient transfer learning and speed up convergence of the target task, we developed a novel mix-up based contrastive learning method to pre-train the ResNet-50 utilized in the CXR-Nets. Parameters in the green network were updated using gradient back propagation, while that in the grey network were updated with momentum update as follows. An infoNCE loss adapted for the momentum update framework was used for training our contrastive learning model, which drove the model to separate different image instances (negative pairs) and same group instances (positive pairs) with data augmentation or image and feature level mix-up. During training of the two CXR-Nets, the learned representations were used to initialize the backbone parameters to achieve advanced prediction performances.


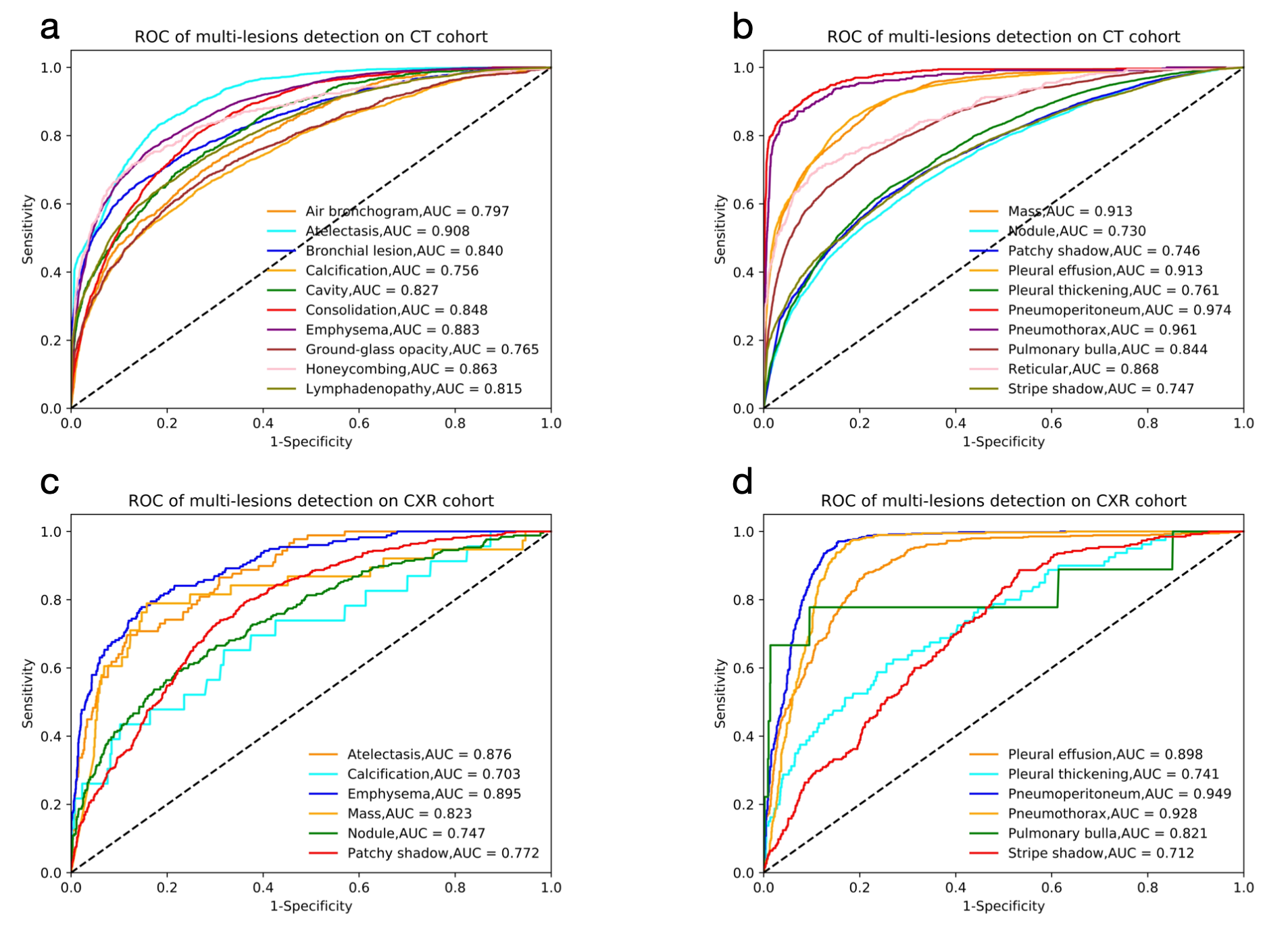


**Supplementary Figure 5. The model performance of the AI system on the external cohort. a-b** ROC curves of AI system of abnormality identification on CT cohort. **c-d** ROC curves of AI system of abnormality identification on CXR cohort.


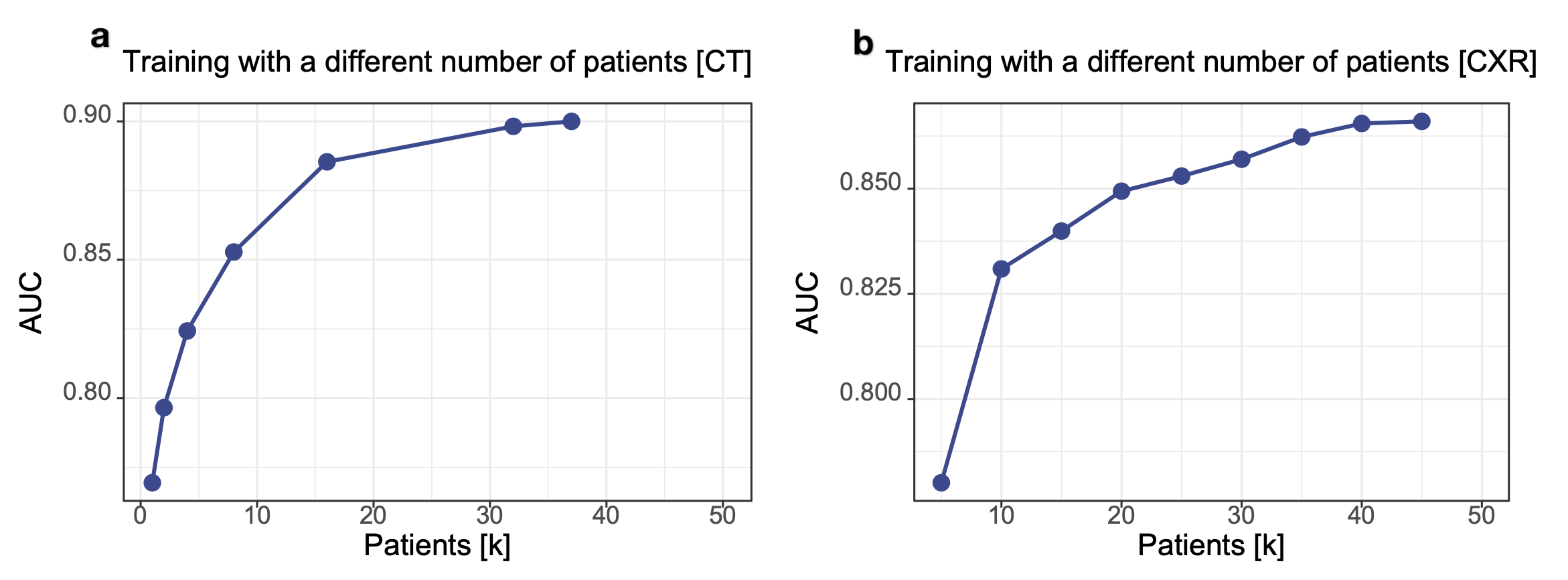


**Supplementary Figure 6. Relationship between model performance with training data scale of included patients in the CT cohort (a) and CXR cohort (b) in the external validation cohort.**


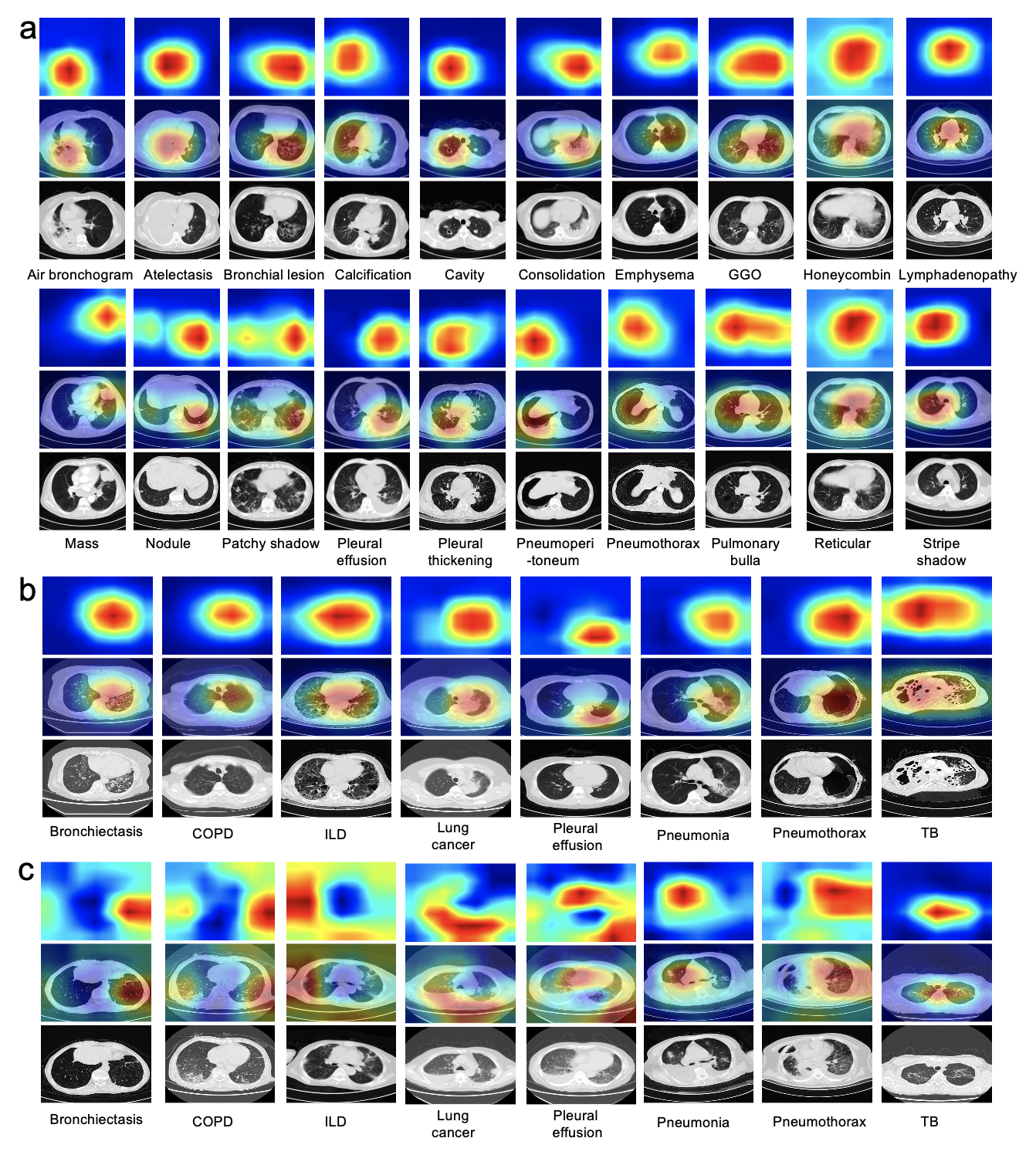


**Supplementary Figure 7. Examples of CT images visualization of features correlated to positivity radiological abnormalities and disease diagnosis.** **a-b** visualizations are given with CAM for correctly classified cases for the abnormality detection and disease diagnosis. **c** visualizations are given with CAM for dubious or misclassified cases. CAM class activation chart. COPD chronic obstructive pulmonary disease, GGO, Ground-glass opacity, ILD interstitial lung disease, TB tuberculosis.


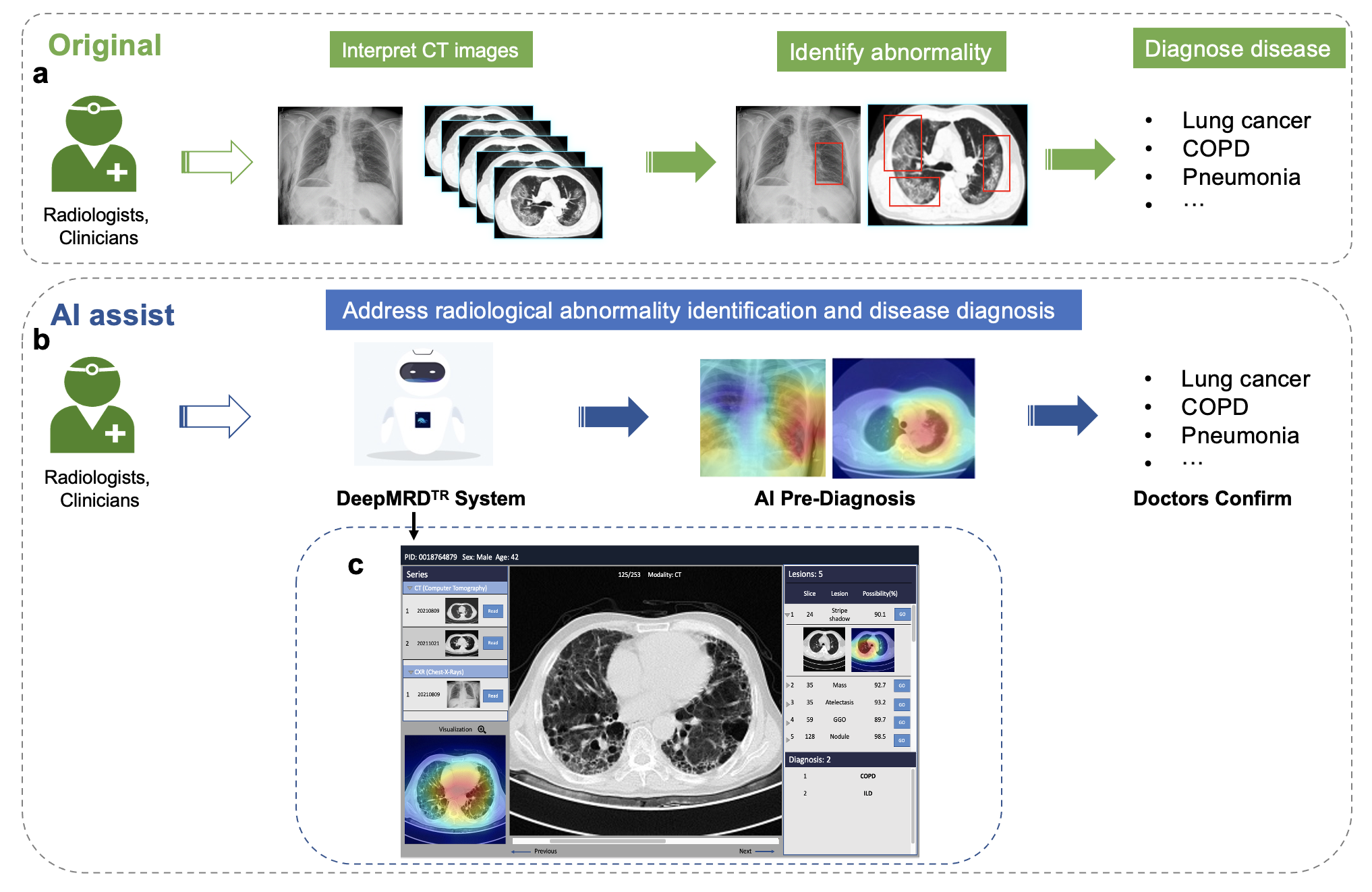


**Supplementary Figure 8. Overview of the process of doctors’ diagnosis of the major respiratory diseases. a** refers to the original schematic of the diagnosis. **b** refers to doctors that use the DeepMRD^TR^ system. **c** shows the interface for doctors while using the diagnostic system.

**Supplementary Tables**

**Supplementary Table 1. Performance of the automatic abnormality label generation module.**

| **Abnormalities** | **Precision** | **Recall** | **F1-score** | **Abnormalities** | **Precision** | **Recall** | **F1-score** |
| --- | --- | --- | --- | --- | --- | --- | --- |
| **Air bronchogram** | 0.95 | 0.92 | 0.84 | **Mass** | 0.88 | 0.94 | 0.93 |
| **Atelectasis** | 0.95 | 0.92 | 0.93 | **Nodule** | 0.92 | 0.98 | 0.95 |
| **Bronchial lesion** | 0.98 | 0.91 | 0.95 | **Patchy shadow** | 0.92 | 0.91 | 0.92 |
| **Calcification** | 0.93 | 0.92 | 0.92 | **Pleural effusion** | 0.94 | 0.96 | 0.95 |
| **Cavity** | 0.94 | 0.99 | 0.94 | **Pleural thickening** | 0.95 | 0.99 | 0.97 |
| **Consolidation** | 0.89 | 0.93 | 0.88 | **Pneumoperitoneum** | 0.96 | 1 | 0.92 |
| **Emphysema** | 1 | 0.96 | 0.98 | **Pneumothorax** | 0.9 | 0.93 | 0.92 |
| **Ground-glass opacity** | 0.98 | 0.97 | 0.98 | **Pulmonary bulla** | 1 | 0.97 | 0.98 |
| **Honeycombing** | 0.95 | 0.99 | 0.91 | **Reticular** | 0.92 | 0.92 | 0.92 |
| **Lymphadenopathy** | 0.89 | 0.95 | 0.92 | **Stripe shadow** | 0.92 | 0.91 | 0.92 |
| **Mean** | 0.94 | 0.95 | 0.93 | **-** | - | - | - |

**Supplementary Table 2. Performance of the automatic disease label generation module.**

| **Diseases** | **Precision** | **Recall** | **F1-score** |
| --- | --- | --- | --- |
| **Bronchiectasis** | 1 | 0.94 | 0.97 |
| **COPD** | 1 | 0.99 | 1 |
| **ILD** | 1 | 0.94 | 0.97 |
| **Lung cancer** | 0.99 | 0.98 | 0.99 |
| **Pleural effusion** | 0.99 | 0.88 | 0.93 |
| **Pneumonia** | 0.98 | 1 | 0.99 |
| **Pneumothorax** | 1 | 0.83 | 0.91 |
| **TB** | 1 | 0.97 | 0.98 |
| **Mean** | 0.99 | 0.94 | 0.97 |

COPD chronic obstructive pulmonary disease, ILD interstitial lung disease, TB tuberculosis.

**Supplementary Table 3. Performance of radiology abnormality detection system based on the CT/CXR cohort.**

| **Abnormalities** | **CT-AUC** | **Sensitivity** | **Specificity** | **CXR-AUC** | **Sensitivity** | **Specificity** |
| --- | --- | --- | --- | --- | --- | --- |
| **Air bronchogram** | 0.820(0.812-0.829) | 0.733(0.715-0.752) | 0.765(0.762-0.770) | 0.811(0.792-0.834) | 0.698(0.658-0.747) | 0.781(0.776-0.786) |
| **Atelectasis** | 0.930(0.927-0.933) | 0.878(0.870-0.885) | 0.846(0.842-0.851) | 0.892(0.882-0.902) | 0.797(0.771-0.827) | 0.838(0.834-0.842) |
| **Bronchial lesion** | 0.850(0.845-0.856) | 0.777(0.765-0.787) | 0.759(0.755-0.765) | - | - | - |
| **Calcification** | 0.772(0.768-0.778) | 0.695(0.686-0.702) | 0.699(0.693-0.704) | 0.756(0.728-0.779) | 0.560(0.502-0.622) | 0.821(0.817-0.826) |
| **Cavity** | 0.856(0.848-0.863) | 0.732(0.715-0.747) | 0.819(0.815-0.823) | 0.866(0.839-0.896) | 0.742(0.655-0.817) | 0.837(0.833-0.841) |
| **Consolidation** | 0.872(0.867-0.876) | 0.819(0.810-0.828) | 0.769(0.764-0.774) | 0.888(0.876-0.898) | 0.853(0.824-0.883) | 0.777(0.773-0.783) |
| **Emphysema** | 0.909(0.906-0.913) | 0.828(0.821-0.836) | 0.830(0.826-0.834) | 0.904(0.897-0.912) | 0.848(0.831-0.867) | 0.805(0.800-0.810) |
| **Ground-glass opacity** | 0.818(0.813-0.825) | 0.702(0.691-0.713) | 0.778(0.773-0.784) | 0.866(0.838-0.897) | 0.718(0.652-0.794) | 0.892(0.889-0.896) |
| **Honeycombing** | 0.915(0.907-0.921) | 0.832(0.814-0.849) | 0.845(0.841-0.849) | 0.915(0.892-0.932) | 0.849(0.783-0.914) | 0.844(0.840-0.848) |
| **Lymphadenopathy** | 0.837(0.833-0.842) | 0.772(0.765-0.780) | 0.749(0.744-0.754) | - | - | - |
| **Mass** | 0.919(0.913-0.925) | 0.850(0.839-0.863) | 0.863(0.860-0.867) | 0.834(0.781-0.870) | 0.606(0.500-0.698) | 0.900(0.897-0.904) |
| **Nodule** | 0.771(0.767-0.776) | 0.709(0.703-0.715) | 0.686(0.676-0.695) | 0.729(0.716-0.741) | 0.599(0.575-0.623) | 0.737(0.732-0.742) |
| **Patchy shadow** | 0.734(0.729-0.739) | 0.708(0.702-0.716) | 0.629(0.622-0.637) | 0.749(0.743-0.755) | 0.657(0.647-0.666) | 0.712(0.707-0.717) |
| **Pleural effusion** | 0.949(0.947-0.952) | 0.883(0.878-0.891) | 0.891(0.886-0.894) | 0.851(0.847-0.856) | 0.800(0.795-0.808) | 0.737(0.731-0.745) |
| **Pleural thickening** | 0.800(0.796-0.806) | 0.747(0.739-0.755) | 0.706(0.700-0.713) | 0.743(0.730-0.756) | 0.707(0.684-0.729) | 0.655(0.649-0.661) |
| **Pneumoperitoneum** | 0.976(0.972-0.981) | 0.904(0.887-0.921) | 0.971(0.969-0.972) | 0.947(0.944-0.951) | 0.899(0.889-0.909) | 0.895(0.891-0.898) |
| **Pneumothorax** | 0.964(0.958-0.971) | 0.900(0.881-0.921) | 0.944(0.942-0.947) | 0.937(0.933-0.942) | 0.901(0.891-0.913) | 0.875(0.870-0.879) |
| **Pulmonary bulla** | 0.883(0.878-0.886) | 0.779(0.769-0.790) | 0.833(0.828-0.838) | 0.944(0.911-0.966) | 0.899(0.837-0.951) | 0.874(0.869-0.877) |
| **Reticular** | 0.922(0.915-0.928) | 0.845(0.830-0.863) | 0.846(0.842-0.850) | 0.912(0.898-0.925) | 0.872(0.828-0.910) | 0.819(0.815-0.825) |
| **Stripe shadow** | 0.737(0.732-0.742) | 0.600(0.593-0.606) | 0.734(0.727-0.741) | 0.736(0.728-0.745) | 0.692(0.677-0.708) | 0.659(0.654-0.665) |
| **Macro AUC** | 0.856(0.843-0.868) | 0.785(0.764-0.804) | 0.790(0.785-0.794) | 0.841(0.832-0.887) | 0.758(0.716-0.799) | 0.792(0.787-0.797) |

**Supplementary Table 4. Performance of multi-disease diagnosis system based on the internal testing CT/CXR cohort.**

| **Pathologies** | **CT-AUC** | **Sensitivity** | **Specificity** | **CXR-AUC** | **Sensitivity** | **Specificity** |
| --- | --- | --- | --- | --- | --- | --- |
| **Bronchiectasis** | 0.885(0.878-0.891) | 0.791(0.780-0.805) | 0.824(0.821-0.828) | 0.814(0.797-0.835) | 0.757(0.727-0.790) | 0.716(0.711-0.719) |
| **COPD** | 0.938(0.935-0.941) | 0.853(0.845-0.859) | 0.870(0.867-0.872) | 0.917(0.913-0.921) | 0.855(0.843-0.866) | 0.809(0.805-0.812) |
| **ILD** | 0.952(0.947-0.956) | 0.857(0.846-0.868) | 0.929(0.926-0.931) | 0.898(0.882-0.913) | 0.808(0.775-0.842) | 0.848(0.845-0.852) |
| **Lung cancer** | 0.952(0.949-0.955) | 0.855(0.846-0.866) | 0.908(0.906-0.910) | 0.952(0.950-0.953) | 0.928(0.924-0.933) | 0.871(0.867-0.874) |
| **Pleural effusion** | 0.916(0.914-0.919) | 0.866(0.861-0.872) | 0.802(0.798-0.805) | 0.854(0.851-0.857) | 0.755(0.747-0.761) | 0.795(0.791-0.799) |
| **Pneumonia** | 0.807(0.803-0.810) | 0.732(0.728-0.735) | 0.737(0.731-0.743) | 0.859(0.855-0.863) | 0.772(0.766-0.777) | 0.832(0.828-0.838) |
| **Pneumothorax** | 0.973(0.970-0.978) | 0.904(0.891-0.917) | 0.954(0.953-0.957) | 0.913(0.908-0.917) | 0.873(0.864-0.883) | 0.812(0.808-0.815) |
| **TB** | 0.890(0.885-0.896) | 0.799(0.788-0.814) | 0.829(0.826-0.833) | 0.812(0.790-0.830) | 0.724(0.680-0.757) | 0.737(0.734-0.741) |
| **Macro AUC** | 0.900(0.872-0.958) | 0.808(0.797-0.821) | 0.848(0.845-0.852) | 0.866(0.832-0.887) | 0.805(0.785-0.824) | 0.786(0.783-0.790) |

COPD chronic obstructive pulmonary disease, ILD interstitial lung disease, TB tuberculosis.

**Supplementary Table 5. Performance of multi-disease diagnosis system based on the external validation CT/CXR cohort.**

| **Pathologies** | **CT-AUC** | **Sensitivity** | **Specificity** | **CXR-AUC** | **Sensitivity** | **Specificity** |
| --- | --- | --- | --- | --- | --- | --- |
| **Bronchiectasis** | 0.881(0.874-0.890) | 0.781(0.760-0.802) | 0.827(0.824-0.831) | 0.798(0.710-0.862) | 0.654(0.480-0.750) | 0.871(0.863-0.880) |
| **COPD** | 0.917(0.915-0.921) | 0.835(0.827-0.844) | 0.842(0.838-0.845) | 0.891(0.857-0.926) | 0.854(0.788-0.910) | 0.813(0.801-0.823) |
| **ILD** | 0.943(0.933-0.954) | 0.860(0.832-0.885) | 0.900(0.897-0.903) | 0.835(0.643-0.980) | 0.875(0.667-1.000) | 0.804(0.791-0.819) |
| **Lung cancer** | 0.920(0.917-0.924) | 0.857(0.846-0.868) | 0.814(0.811-0.818) | 0.893(0.885-0.903) | 0.897(0.881-0.913) | 0.739(0.727-0.751) |
| **Pleural effusion** | 0.877(0.873-0.881) | 0.739(0.729-0.748) | 0.821(0.816-0.824) | 0.822(0.806-0.836) | 0.792(0.766-0.823) | 0.707(0.692-0.719) |
| **Pneumonia** | 0.807(0.803-0.811) | 0.795(0.787-0.803) | 0.684(0.679-0.688) | 0.802(0.783-0.819) | 0.672(0.637-0.708) | 0.790(0.778-0.803) |
| **Pneumothorax** | 0.961(0.956-0.967) | 0.875(0.854-0.895) | 0.923(0.920-0.925) | 0.892(0.883-0.902) | 0.939(0.926-0.953) | 0.687(0.670-0.700) |
| **TB** | 0.874(0.862-0.887) | 0.801(0.776-0.828) | 0.773(0.768-0.776) | 0.862(0.812-0.901) | 0.757(0.640-0.861) | 0.849(0.838-0.857) |
| **Macro AUC** | 0.882(0.825-0.908) | 0.807(0.786-0.826) | 0.804(0.800-0.807) | 0.841(0.801-0.884) | 0.811(0.733-0.869) | 0.761(0.748-0.773) |

COPD chronic obstructive pulmonary disease, ILD interstitial lung disease, TB tuberculosis.
