# Supplementary material for: Development and validation of an abnormality-derived deep-learning diagnostic system for major respiratory diseases
Source: NPJ Digit Med. 2022 Aug 23;5:124. doi: 10.1038/s41746-022-00648-z (PMC9395860; doi:10.1038/s41746-022-00648-z)
Supplement: Supplementary file 2 — Reporting Summary [file 41746_2022_648_MOESM2_ESM.pdf]

nature portfolio

Corresponding author(s): Weimin Li

Last updated by author(s): Feb 23, 2022

Reporting Summary

Nature Portfolio wishes to improve the reproducibility of the work that we publish. This form provides structure for consistency and transparency in reporting. For further information on Nature Portfolio policies, see our Editorial Policies and the Editorial Policy Checklist.

Statistics

For all statistical analyses, confirm that the following items are present in the figure legend, table legend, main text, or Methods section.

n/a	Confirmed	
☐	☒	The exact sample size (n) for each experimental group/condition, given as a discrete number and unit of measurement
☒	☐	A statement on whether measurements were taken from distinct samples or whether the same sample was measured repeatedly
☒	☐	The statistical test(s) used AND whether they are one- or two-sided<br>*Only common tests should be described solely by name; describe more complex techniques in the Methods section.*
☒	☐	A description of all covariates tested
☒	☐	A description of any assumptions or corrections, such as tests of normality and adjustment for multiple comparisons
☐	☒	A full description of the statistical parameters including central tendency (e.g. means) or other basic estimates (e.g. regression coefficient) AND variation (e.g. standard deviation) or associated estimates of uncertainty (e.g. confidence intervals)
☐	☒	For null hypothesis testing, the test statistic (e.g. F, t, r) with confidence intervals, effect sizes, degrees of freedom and P value noted<br>*Give P values as exact values whenever suitable.*
☒	☐	For Bayesian analysis, information on the choice of priors and Markov chain Monte Carlo settings
☒	☐	For hierarchical and complex designs, identification of the appropriate level for tests and full reporting of outcomes
☐	☒	Estimates of effect sizes (e.g. Cohen's d, Pearson's r), indicating how they were calculated

Our web collection on statistics for biologists contains articles on many of the points above.

Software and code

Policy information about availability of computer code

Data collection	No special software or code was used to collect the data
Data analysis	Pytorch and python libraries

For manuscripts utilizing custom algorithms or software that are central to the research but not yet described in published literature, software must be made available to editors and reviewers. We strongly encourage code deposition in a community repository (e.g. GitHub). See the Nature Portfolio guidelines for submitting code & software for further information.

Data

Policy information about availability of data

All manuscripts must include a data availability statement. This statement should provide the following information, where applicable:

- Accession codes, unique identifiers, or web links for publicly available datasets
- A description of any restrictions on data availability
- For clinical datasets or third party data, please ensure that the statement adheres to our policy

The raw image dataset generated or analysed during the current study are not publicly available due to the DICOM metadata containing information that could comprise patient privacy/consent. The main data supporting the results in this study are available within the paper and its Supplementary Information. Please email all requests for academic use of raw and processed data to the corresponding author (and the copy to weimi003@scu.edu.cn). All requests will be evaluated based on institutional and departmental policies to determine if the data requested is subject to intellectual property or patient privacy obligations. Data can only be shared for non-commercial academic purposes and require a formal material transfer agreement.

Field-specific reporting

Please select the one below that is the best fit for your research. If you are not sure, read the appropriate sections before making your selection.

☒ Life sciences ☐ Behavioural & social sciences ☐ Ecological, evolutionary & environmental sciences

For a reference copy of the document with all sections, see nature.com/documents/nr-reporting-summary-flat.pdf

Life sciences study design

All studies must disclose on these points even when the disclosure is negative.

Sample size	We constructed a large chest scan dataset from two primary subsets: one from West China Hospital (WCH) for training and in-house validation, and the other from Chengdu ShangJin Nanfu Hospital (CSJH) for external testing. Of 228,563 CT volumes (n=52,220) came from two hospitals, 191,333 volumes were chosen randomly for the development of the AI system, while 37,230 volumes for external validation. A total of 129,319 images (n=67,611) were simultaneously included for the same tasks, of which 125,599 CXR images were used for training and validation, and an additional 3,720 instances were added for extra-house validation.
Data exclusions	The data exclusion criteria was pre-established. We excluded data based on the following criteria: (a) having only one post-operative image; (b) being diagnosed with other rare diseases other than the eight major respiratory diseases we defined; (c) being under the age of 16; (d) having radiological studies with image reconstruction kernels unrelated to the lung and view positions unrelated to the chest (e.g., only AP/PA were reserved); or having views with motion artifacts.
Replication	Replication was not relevant. We used independent validation cohorts.
Randomization	Samples were randomly allocated to the training, tuning and testing sets.
Blinding	All images were de-identified during image processing to remove any patient-related information.

Reporting for specific materials, systems and methods

We require information from authors about some types of materials, experimental systems and methods used in many studies. Here, indicate whether each material, system or method listed is relevant to your study. If you are not sure if a list item applies to your research, read the appropriate section before selecting a response.

Materials & experimental systems

n/a	Involved in the study	
☒	☐	Antibodies
☒	☐	Eukaryotic cell lines
☒	☐	Palaeontology and archaeology
☒	☐	Animals and other organisms
☐	☒	Human research participants
☒	☐	Clinical data
☒	☐	Dual use research of concern

Methods

n/a	Involved in the study	
☒	☐	ChIP-seq
☒	☐	Flow cytometry
☒	☐	MRI-based neuroimaging

Human research participants

Policy information about studies involving human research participants

Population characteristics	Images were obtained as a part of routine clinical care.
Recruitment	No patient recruitment was performed. All present CT/CXR images and associated clinical information that were available for the preestablished collecting period were analyzed.
Ethics oversight	Institutional Review Board (IRB)/Ethics Committee of West China Hospital (WCH) and Chengdu ShangJin Nanfu Hospital (CSJH).

Note that full information on the approval of the study protocol must also be provided in the manuscript.
